# Pyramidal cell axon initial segment in Alzheimer´s disease

**DOI:** 10.1038/s41598-022-12700-9

**Published:** 2022-05-24

**Authors:** Alejandro Antón-Fernández, Gonzalo León-Espinosa, Javier DeFelipe, Alberto Muñoz

**Affiliations:** 1 Department of Functional and Systems Neurobiology, Instituto Cajal, CSIC, Madrid, Spain; 2 Laboratorio Cajal de Circuitos Corticales (CTB), Universidad Politécnica de Madrid, Madrid, Spain; 3 CIBERNED, Centro de Investigación Biomédica en Red de Enfermedades Neurodegenerativas, Madrid, Spain; 4 Department of Cell Biology, Universidad Complutense de Madrid, Madrid, Spain; 5 Departamento de Química y Bioquímica, Facultad de Farmacia, Universidad San Pablo-CEU, CEU Universities, Urbanización Montepríncipe, 28660 Boadilla del Monte, Madrid, Spain; 6 Present Address: Department of Molecular Neuropathology, Centro de Biología Molecular Severo Ochoa, CBMSO, CSIC-UAM, Madrid, Spain

**Keywords:** Cell biology, Neuroscience

## Abstract

The axon initial segment (AIS) is a region of the neuron that is critical for action potential generation as well as for the regulation of neural activity. This specialized structure—characterized by the expression of different types of ion channels as well as adhesion, scaffolding and cytoskeleton proteins—is subjected to morpho-functional plastic changes in length and position upon variations in neural activity or in pathological conditions. In the present study, using immunocytochemistry with the AT8 antibody (phospho-tau S202/T205) and 3D confocal microscopy reconstruction techniques in brain tissue from Alzheimer’s disease patients, we found that around half of the cortical pyramidal neurons with hyperphosphorylated tau showed changes in AIS length and position in comparison with AT8-negative neurons from the same cortical layers. We observed a wide variety of AIS alterations in neurons with hyperphosphorylated tau, although the most common changes were a proximal shift or a lengthening of the AISs. Similar results were found in neocortical tissue from non-demented cases with neurons containing hyperphosphorylated tau. These findings support the notion that the accumulation of phospho-tau is associated with structural alterations of the AIS that are likely to have an impact on normal neuronal activity, which might contribute to neuronal dysfunction in AD.

## Introduction

The axon initial segment (AIS) is the cellular domain in neurons which is characterized by a unique architecture as well as molecular and functional specializations and which occupies around 20–60 μm of the proximal axon length^1^. Therefore, it represents a small, intermediate region of the axon between the somatodendritic domain and distal axonal compartments. Due to the contribution of the AIS to the generation and modulation of action potentials, owing to the high concentration of ion channels situated there^2,3^, AIS structural changes have important consequences for network excitability^2,4^. In fact, it has been shown that plastic changes in the AIS microanatomy occur in response to under- or over-stimulation of neurons, adjusting the excitability of a neuron in response to its inputs, which contributes to the homeostasis of neuronal circuits^5,6^. Specifically, properties of the AIS such as length or position (i.e., point where the AIS originates in the axon hillock, at the base of the cell body or a main dendrite) can determine the firing properties of neurons^4,7^. For example, an increase in the AIS length in neurons from chicken brainstem auditory nuclei has been reported after loss of auditory input due to cochlea removal^6^. Also, it has been shown that the depolarization of dentate granule cells shortened their AIS^8^, whereas—in CA1 pyramidal neurons—depolarization promoted a distal AIS shift^9^, decreasing neuronal excitability in both cases.

The importance of the AIS has also been demonstrated by the involvement of its alterations in distinct pathological conditions such as demyelination, traumatic brain injury, Angelman syndrome, stroke, schizophrenia, autism, epilepsy and channelopathies^10–17^. The involvement of AIS alterations in Alzheimer’s disease (AD) patients has not been directly examined despite the fact that AD is the most common neurodegenerative disorder and alterations of the AIS have been reported in mouse models of the disease. The main pathological hallmarks of AD are the presence of senile plaques made of extracellular deposits of amyloid-β peptides and intracellular neurofibrillary tangles of hyperphosphorylated tau. Using the APP (amyloid precursor protein) transgenic mouse model of AD, it has been found that the presence of Aβ plaques induced an impairment in GABAergic innervation of the AIS^18^ and also leads to a reduction in AIS length and βIV-Spectrin expression, which is an essential structural protein of AIS^19^.

Regarding tau hyperphosphorylation, previous studies revealed that alterations of the barrier functions of the AIS—that restrict various isoforms of tau to specific neuronal domains^20–23^—are disrupted in AD mouse models resulting in the missorting of hyperphosphorylated tau, which accumulates in the somatodendritic domain^24^. In vitro studies have shown that tau phosphorylation or the downregulation of AIS proteins like Ankyrin G or EB1 induce the missorting of tau by disruption of the AIS diffusion barrier, which has been suggested to participate in the pathogenesis of AD^24–26^. The presence of phosphorylated tau in the somatodendritic compartment has also been shown in vitro to decrease neuronal activity^27,28^, which was correlated with an AIS distal shift along the axon^29^. The expression of AIS cytoskeletal proteins such as AnkyrinG or βIV-Spectrin, which are essential for AIS integrity maintenance^30,31^, has been found to be downregulated in AD brains^32^, which could conceivably be related to plastic changes or pathological alterations of the AIS. However, the possible structural alterations of the AIS in cortical neurons from AD patients, and their possible relationship with phospho-tau accumulation, have not been directly explored. In the present study, we address the potential impact that the presence of phospho-tau aggregates might have on AIS integrity in neocortical tissue from AD patients. In order to label neurons that accumulate hyperphosphorylated tau, we used antibody AT8 since as previously discussed^33^ phosphorylation at Ser202/Thr205 (epitope recognized by the antibody AT8) represents an early degenerative change of the cytoskeleton^34,35^, which plays a central role in the successive tau phosphorylation ^36^ and has been associated with early stages of the AD^37,38^. AT8 is used to classify the neurofibrillary degeneration into stages known as the Braak stages^39^.The results indicate that the presence of somatodendritic phospho-tau is associated with variety of alterations in the length and/or position of the AIS, which might contribute to changes in the activity of neuronal circuits in tauopathies.

## Results

**Figure 1:**
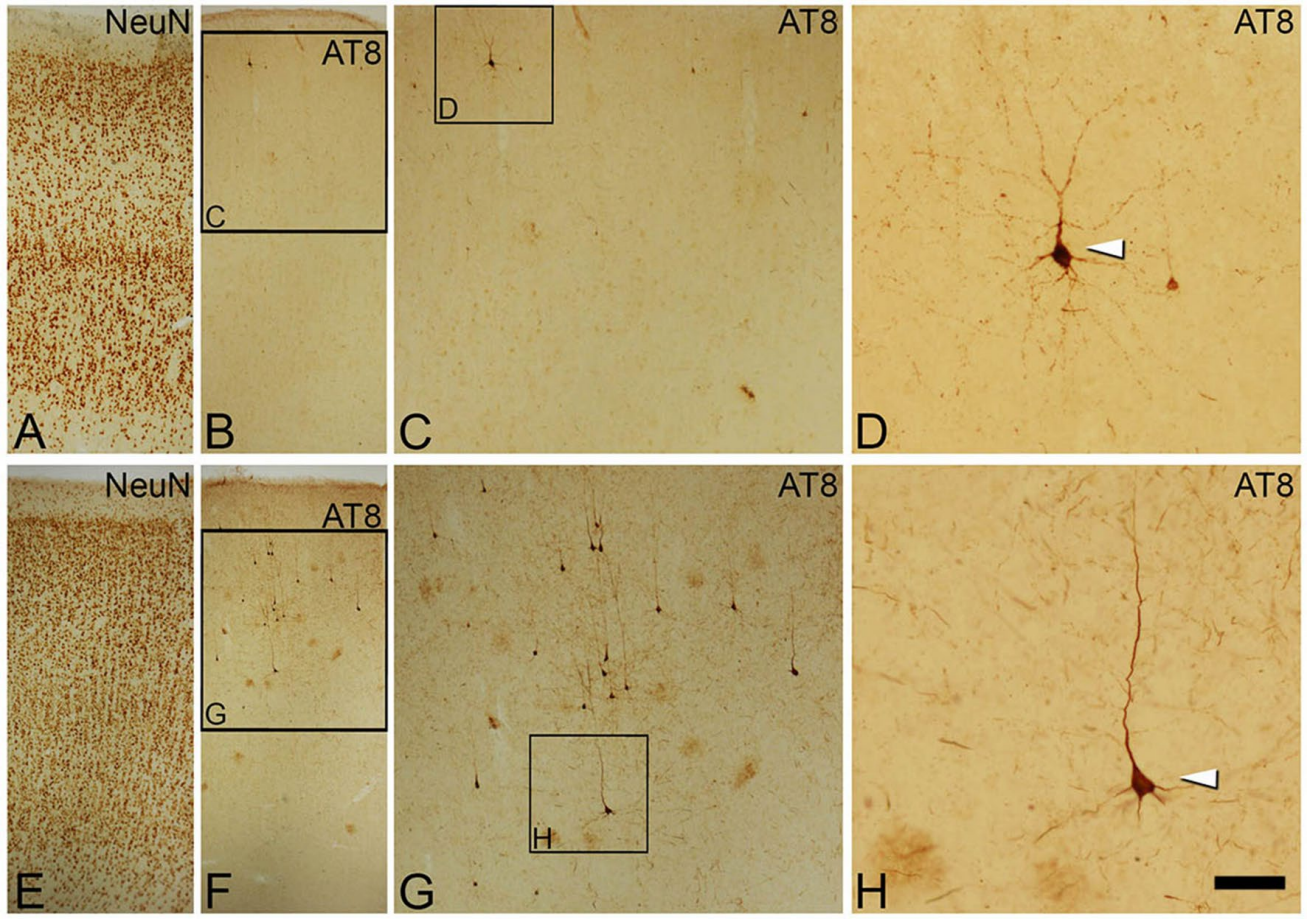
Photomicrographs taken from brain sections of the temporal neocortex from an AD patient (IF8, **A**−**D**) and a non-demented human case (AB1, **E**−**H**), showing the distribution of neurons with hyperphosphorylated tau revealed by DAB immunostaining with AT8 antibodies (**B** and **F**) in different cortical layers identified by NeuN immunostaining of adjacent sections (**A** and **E**, respectively).Squared areas in B, C, F and G are shown at higher magnification in C, D, G and H, respectively. Arrowheads indicate AT8-positive neurons. Scale bar shown in H indicates 106 µm in A, B, E and F, 47 µm in C and G and 21 µm in D and H. Adobe Photoshop CS4 software was used to compose figures.

In order to study the possible relationship between the accumulation of hyperphosphorylated tau in neurons and the structural alterations of the AIS, we performed experiments with triple immunocytochemical staining of neocortical tissue from two sources: from autopsies of AD patients and from autopsy of individuals that were free of any known neurological or psychiatric illness, but with a high neurofibrillary tangles load (cases AB1 and AB6, see Table 1) that were considered controls. In all cases, we combined antibodies against βIV-Spectrin and NeuN, to label the AIS and neuronal cell body, respectively, and AT8 antibodies to label neurons that accumulate hyperphosphorylated tau (phospho-Ser202 and phosho-Thr205) (Fig. 1). We have analyzed the possible alterations of the AISs in all AT8 positive neurons compared to neighbor AT8 negative neurons of the same individual, neuronal region and cortical layer.

**Table 1:** Summary of clinical data and neurologic diagnosis according to Braak and Braak criteria^40^, defined by different stages (from I to VI), and according to CERAD criteria (Consortium to Establish a Registry for Alzheimer’s Disease)^41^, which use a semiquantitative score of the density of neuritic plaques in the most severely affected region of the isocortex (A = mild presence of plaques, B = moderate presence of plaques, C = severe presence of plaques).

Cases	Age (years)	Gender	Postmortem delay (hours)	Neurologic diagnosis	Additional Neurological diagnosis	Cause of death
IF6	85	Male	2	AD III-A	–	–
IF8	91	Male	2.5	AD III-A	AGD stage III, neuronal ballooning	–
IF13	75	Male	2	AD III-B	AGD stage III	Lymphoproliferative disorder
AB1	45	Male	< 1	–	–	Pleural *mesothelioma*
AB6	92	Female	4	III-A	Aging-related tau astrogliopathy	–
IF1	80	Female	2	AD IV-B	–	–
IF5	80	Female	3	AD III, –	TDP43	–
IF7	88	Female	2	AD III, –	AGD	–
AB5	59	Male	4	III, –	–	–
Vk11	87	Female	1.5	AD III/IV—A	Small Vessel Disease	Respiratory infection
Vk16	88	Female	2	AD VI-C	Vascular alterations; LBD; HS	–
Vk22	86	Female	2	AD V	Amyloid angiopathy. Small Vessel Disease. Vessel atheromatosis	–
VK27	95	Female	4	AD V-B	LBD (amygdala) Type II Amyloid angiopathy	–
Bcn2	82	Female	2	AD V-C	α Syn (amygdala); HS	–
Bcn3	81	Female	5.30	AD V-C	TDP43	–
Bcn5	83	Female	4	AD V/VI-C	Microinfarctions	–
Bcn8	64	Female	< 6	AD VI-C	Amyloid angiopathy	–
Bcn12	74	Female	3.30	AD VI-C	Small Vessel Disease	–

We have used an internal code to ensure the confidentiality of each sample. Red indicates those cases in which antibodies to βIV-Spectrin labeled the AISs. AGD, Argyrophilic grain disease; LBD, Lewy body disease; HS, hippocampal sclerosis; Syn, synuclein; TDP43, TAR DNA-binding protein 43; –, not available. Cases IF6, IF8, IF13 were AD cases, whereas AB1 and AB6 were free of any known neurological or psychiatric illness (considered as controls).

### Alterations of the AIS in neurons accumulating phosphorylated tau in AD and control cases

**Figure 2:**
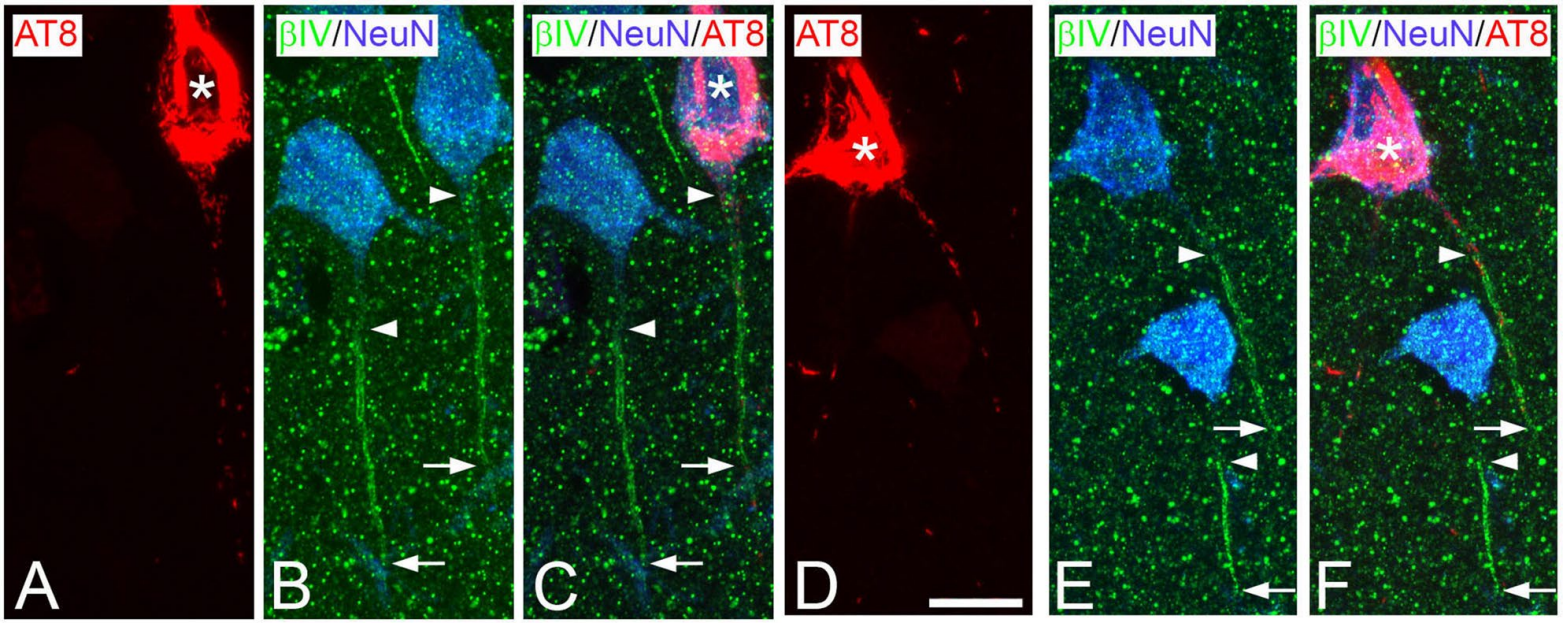
Trios of confocal stack Z projection microphotographs taken from AT8 (red)/βIV-Spectrin (green)/NeuN (blue) triple-immunostained sections from the temporal neocortex of an AD patient (IF13, **A**−**C**) and from a non-demented case (AB1, **D**−**F**). Arrowheads and arrows indicate the start point and the end point, respectively, of AISs labeled by βIV-Spectrin immunostaining. Asterisks indicate neurons containing neurofibrillary tangles. Scale bar shown in D indicates 11 µm in **A**−**F**. Adobe Photoshop CS4 (Adobe Inc., 2019) software was used to compose figures.

A limitation of the present study is the relatively low number of AISs analyzed. Several factors contribute to this limitation. To our knowledge, the rabbit anti-βIV-Spectrin antibodies used are among the best options to label AIS in human brain tissue in combination with mouse AT8 antibody, which detects phosphorylated-tau at positions S205 and T205. However, among the 18 tested cases available in our laboratory (Table 1), some of which had been successfully used in previous studies using different antibodies^42^; βIV-Spectrin antibodies only labeled AISs in tissue from three AD patients (IF6, IF8 and IF13) and in two non-demented cases (AB1 and AB6), all of which had postmortem delay times below 4 h. In addition, in two (IF6 and IF8) out of these cases, the presence of neurons immunostained with AT8 antibodies was relatively low (Fig. 1), reducing the potential number of AISs to study. It is also known that contact with—or close proximity of—β-amyloid plaques induces structural alterations in the AIS^18,19^. Therefore, to investigate the possible effects on the AIS caused by the presence of the aggregation of hyperphosphorylated tau (minimizing the effects induced by the proximity of β-amyloid plaques), we focused only on AISs from pyramidal neurons that were non-adjacent to β-amyloid plaques, which were AT8-immunopositive or AT8-immunonegative. In these regions, distant from β-amyloid plaques, we only considered for analysis the AIS from those neocortical pyramidal neurons whose cell bodies and AISs were completely included within the tissue thickness in which both AT8 and βIV-Spectrin antibodies penetrated. This rendered patterns of AIS βIV-Spectrin immunoreactivity (Fig. 2) apparently similar to those found in human control tissue in previous studies from our laboratory^43^.

**Figure 3:**
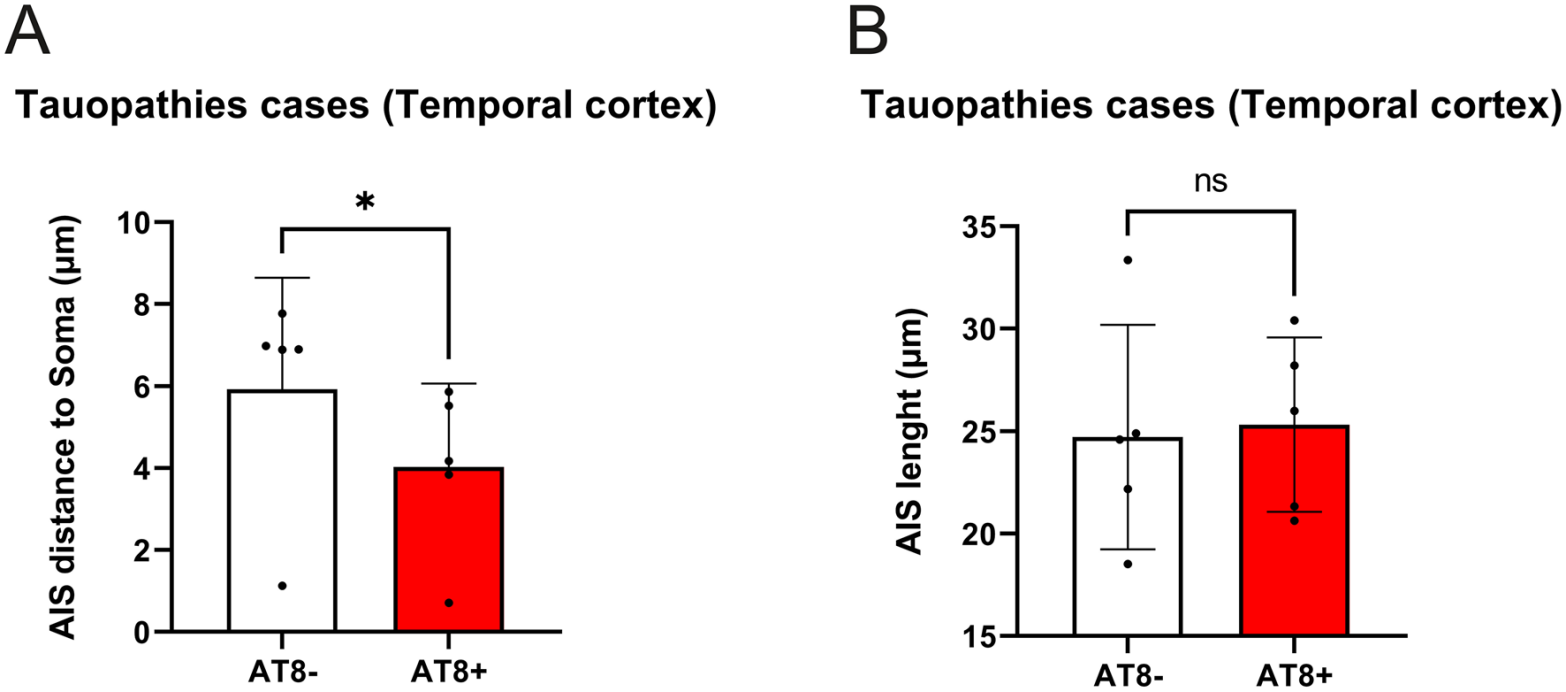
Histograms showing the comparison of AIS distance to soma (**A**) and the AIS length (**B**) between all the AT8- and AT8 + neurons analysed. Note the significant (*p *= 0.02) decrease in AIS distance to soma in AT8 + neurons (SPSS software). Adobe Photoshop CS4 software was used to compose figures. Imaris software was used to measure—in 3D—the AIS length and/or position.

In a first general analysis of the possible AIS alterations, we collectively analyzed values in AT8-immunopositive neurons obtained in AD and control cases (n = 5). Taking mean values of AIS length and distance from the soma (354 neurons), we found statistically significant differences (*p* = 0.02) in the AIS position between AT8-immunopositive (mean ± SD; 4.026 ± 2.04 μm) and AT8-immunonegative (5.93 ± 2.71 μm). The AIS in AT8-immunopositive neurons was found to be closer to the soma than in surrounding neurons without phospho-tau aggregates, whereas we found no statistically significant differences in AIS length (Fig. 3).

**Figure 4:**
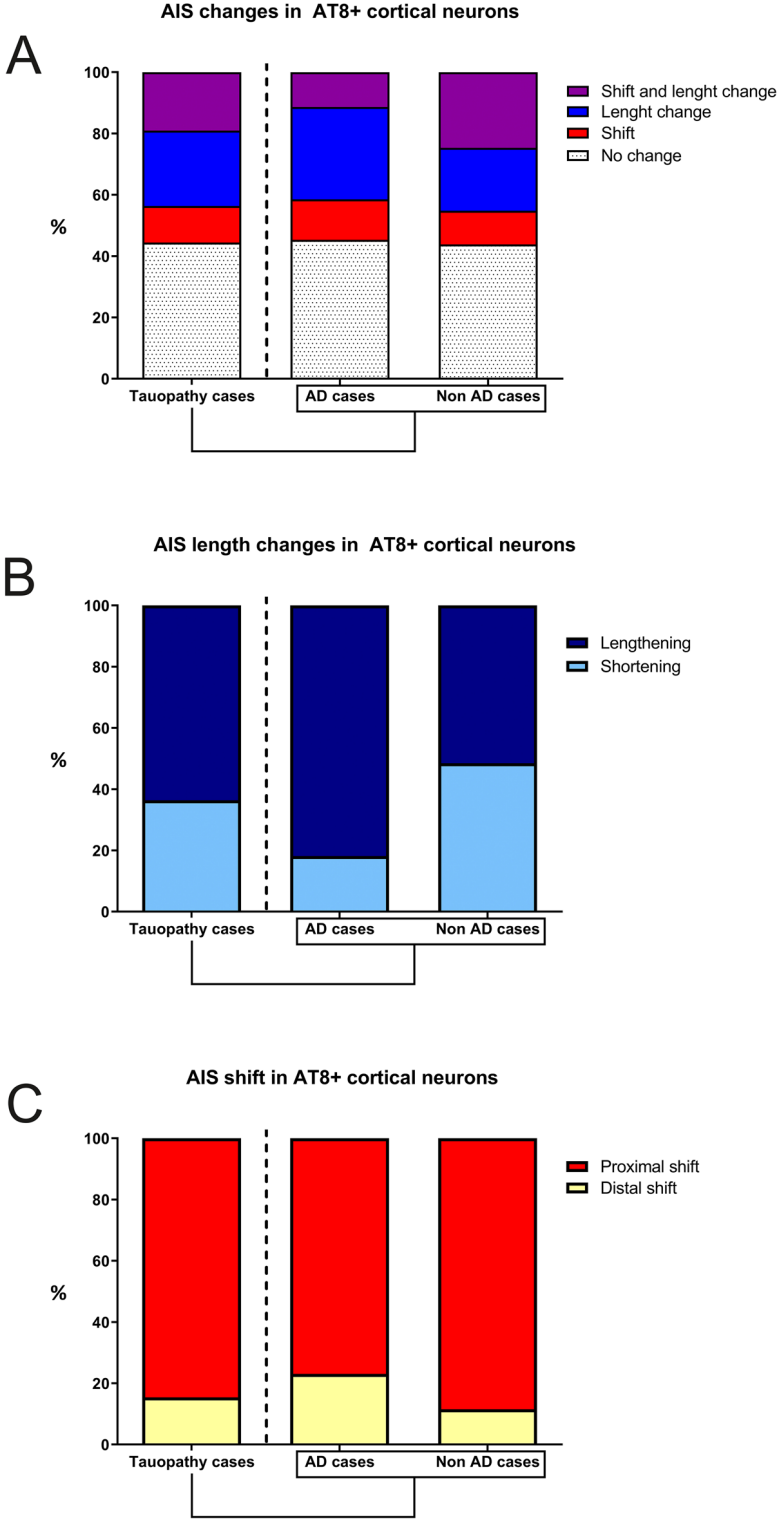
Histograms showing—in all tauopathy cases (left column), in AD cases (middle column) and in control cases (right column)—the percentages of AT8-immunopositive neocortical neurons with different AIS changes (**A**), with different AIS length changes (**B**) and with different types of AIS shift (**C**). Adobe Photoshop CS4 software was used to compose figures. Imaris software was used to measure—in 3D—the AIS length and/or position.

In the tissue analyzed from each case, we found different numbers of AT8-positive neurons across cortical layers (Table 2). An important limitation when addressing the AIS structural alterations caused by the accumulation of hyperphosphorylated tau is that it is not possible to know the AIS length and position in AT8-immunopositive neurons prior to the phospho-tau accumulation. Furthermore, it is known that there is considerable variability in AIS length between neurons in different cortical layers and between different neuronal populations^44,45^. Therefore, in order to avoid inter-individual and inter-layer variability, we further compared, AIS length and position data from every AT8-immunopositive neuron with mean ± standard deviation values obtained from all AT8-immunonegative neurons in the same cortical layer. We considered that an AT8-immunopositive was altered when its differences in AIS length and/or position values, with the corresponding mean values, exceeded the standard deviation. Using this one-by-one analysis, we found that 70 out of 126 (55.6%) AT8-immunopositive neurons from all tauopathy cases (AD and controls together) showed changes in AIS length and/or position as compared to AT8-immunonegative neurons (Fig. 4A, Table 3). AIS length and position were unaltered in the remaining 44.4% of AT8-immunopositive neurons, despite the presence of tau hyperphosphorylation (Fig. 4A). Furthermore, we found marked heterogeneity in the AIS length and position changes in AT8-immunopositive neurons. In 63.6% (35 out of 55) of the AT8-immunopositive neurons with a change in AIS length, the AIS was longer (9.4 ± 3.5 μm) than in AT8-immunoneagative neurons, whereas it was shorter in the remaining 36.4% (Fig. 4B). We also found that, in 84.6% (33 out of 39) of the AT8-immunopositive neurons with a change in AIS position, the AIS start point was located closer to the soma (proximal shift of 3.6 ± 1.41 μm) than in AT8-immunonegative neurons, whereas it was located further down the axon only in the remaining 12.8% (Fig. 4C). Finally, some of the AT8-immunopositive neurons (24 out of 126, 19%) showed simultaneous changes (in the same neuron) in AIS length and position.

**Table 2:** Table showing the number of neurons and mean values for AIS length and AIS distance to soma in AT8-immunopositive or AT8-immunonegative in the different cortical layers in each case.

	AD cases	Control cases
AT8 + neurons in Layer II (n/AIS length/AIS distance to soma)	28/27.4/5.9	12/25.8/2.4
AT8 + neurons in Layer III (n/AIS length/AIS distance to soma)	22/27.6/5.4	23/27.9/2.5
AT8 + neurons in Layer IV (n/AIS length/AIS distance to soma)	9/27.7/6.1	7/27.5/7
AT8 + neurons in Layer V (n/AIS length/AIS distance to soma)	16/22/4.1	47/27/5.2
Total number of AT8 + / AT8- neurons	75/120	89/70
Total number of neurons examined	195	159
AIS mean length in AT8 + neurons	26.3	27.13
AIS mean length in AT8- neurons	23.8	26.73
AIS mean distance to soma in AT8 +	5.4	4.2
AIS mean distance to soma in AT8-	6.9	6.1

Length and distance are expressed in µm. Mean values are calculated considering all individual neurons.

**Table 3:** Table showing percentage values of AT8-immunopositive neurons with AIS length and/or position changes in compared to AT8-immunonegative neurons in AD and control cases.

	AD cases	Control cases
AT8 + neurons with AIS changes	(29 out of 53) 54.7%	(41 out of 73) 56.2%
AT8 + neurons with no AIS changes	(24 out of 53) 45.3%	(32 out of 73) 43.8%
AT8 + neurons with AIS changes in AIS length and position	(6 out of 53) 11.3%	(18 out of 73) 24.7%
AT8 + neurons with changes only in AIS position	(7 out of 53) 13.2%	(8 out of 73) 11%
AT8 + neurons with AIS proximal shift	(10 out of 13) 76.9%	(23 out of 26) 88.5%
AT8 + neurons with AIS distal shift	(3 out of 13) 23.1%	(3 out of 26) 11.5%
AT8 + neurons with changes only in AIS length	(16 out of 53) 30.2%	(15 out of 73) 20.5%
AT8 + neurons with AIS lengthening	(18 out of 22) 81.8%	(17 out of 33) 51.5%
AT8 + neurons with AIS shortening	(4 out of 22) 18.2%	(16 out of 33) 48.5%

Note that some AT8-immunopositive neurons may contain more than one type of AIS change at the same time.

To test the possible AIS variations in AD, the same one-by-one analysis was performed just in the AD cases. Our results revealed that 54.7% (29 out of 53) of the AT8-immunopositive neurons showed changes in AIS length and/or position as compared to AT8-immunonegative neurons (Fig. 4, Table 3). AIS length and position were unaltered in the remaining 44.4%. It is noteworthy that the most frequent changes were a proximal shift (76.9%), being the AIS start point located closer to the soma (3.8 ± 0.8 µm) and an AIS lengthening (81.8%), with an average of 8.6 ± 2.6 μm of AIS expansion). These results are in agreement with the AIS changes found in the above—all tauopathy cases—analysis.

Finally, to test the potential effects induced by the sole presence of aggregates of hyperphosphorylated tau, presumably without any other potential pathologic factor such as β-amyloid pathology, we performed the same analysis in the two cases with no recorded neurological or psychiatric alterations (AB1 and AB6). In these cases, we found a relatively large number of AT8-immunopositive neurons in the temporal cortex (Fig. 1, E−H) in the absence of β-amyloid plaques. The analysis revealed that, in more than half of all AT8-immunopositive neurons (56.2%), AIS length and/or position values were different to those of AT8-immunonegative neurons. AIS length and position were unaltered in the remaining 43.8% of AT8-immunopositive neurons, despite the presence of hyperphosphorylated tau (Fig. 4A). In addition, we found that in 51.5% of the AT8-immunopositive neurons with a change in AIS length, the AIS was longer (10.01 ± 4.8 µm) than in AT8-immunonegative neurons, whereas it was shorter (7.09 ± 3.23 µm) in the remaining 48.5% (Fig. 4B). We also found that in all the AT8-immunopositive neurons with a change in AIS position, the AIS start point was located closer to the soma (proximal shift of 4.21 ± 1.63 µm) than in AT8-immunonegative neurons (Fig. 4C). In summary, our results show an important heterogeneity of AIS changes in neurons accumulating phosphorylated tau, being AIS proximal shift and lengthening, the most frequent observations.

## Discussion

The importance of AIS structural alterations due to their potential involvement in many psychiatric and neurological disorders has been previously recognized (see^11^). In the case of AD, downregulation of the AIS proteins AnkyrinG or βIV-Spectrin, which are essential for the maintenance of AIS integrity^30,31,46^, was found in AD patients at late (V−VI)- as compared to early (I−II)-Braak stages^32^. In line with the study by Sohn et al.^32^, in the present study we found that, βIV-Spectrin immunostaining of the AIS was preserved in cortical tissue from AD patients only at relatively early stages of the disease (stage III). By contrast, no βIV-Spectrin AIS immunostaining was found in tissue from twelve AD patients that were, in all but two cases, at more advanced stages of the disease (Table 1).

In addition to ante-mortem factors (age, use of toxic substances and drugs, duration of agonal state, etc.), it is known that a major limitation in studies using brain tissue from autopsy is that various post-mortem factors affect histological stainings. Among the most critical factors are post-mortem time, tissue fixation and staining procedures^40^. In the present study, since there were no differences in these parameters between patients, and the post-mortem delay times in most cases (all but one) were below 5 h, known to be optimal for histological staining^47^, the differences observed in βIV-Spectrin immunostaining of the AIS might be considered to relate to the disease. The lack of βIV-Spectrin immunostaining in tissue from the AD patients with advanced stages could be partially due to the presence of Aβ plaques. For example, a decrease in AIS length and βIV-Spectrin expression has been shown in APP-PS1 mouse model of amyloidosis^19^, in addition to alterations in AIS GABAergic innervation in the same animal model^18^. However, our results indicate that the plaque load is probably not a critical factor responsible for the decrease in AIS βIV-Spectrin immunostaining observed in AD, since this staining was preserved in a patient with a relatively high plaque load (case IF13).

In the present study, we have used the AT8 antibody to detect hyperphosphorylated tau. The epitope recognized by this antibody (tau phosphorylation in Ser202/Thr205) has been proposed as an early event for microtubule destabilization in AD brains, participating in the sequential phosphorylation of the tau protein^35^. However, AT8 antibody also recognizes intraneuronal and extracellular tau aggregates in more advanced states^48,49^. In a recent study with lower Braak stage cases (I-III), it has been reported that almost 80% of cells labelled with pS396 (a late phospho-tau marker in AD) colocalizes with the AT100 antibody^33^. The appearance of AT100 epitope, in the sequential tau phosphorylation progression, is similar to AT8, both present in extracellular NFTs^50,51^. Therefore, the number of AT8- cells with phospho-tau accumulation in these low Braak stage human cases is expected to be low. However, further studies using new antibodies directed to AIS markers, in combination with antibodies that recognize tau that is phosphorylated at additional residues, will be helpful to gain deeper insight into AIS vulnerability to the progression of tau hyperphosphorylation during the course of AD.

Studies in AD mouse models have shown that the barrier functions of the AIS—that control the traffic of tau and help restrict various isoforms of tau to specific neuronal domains—are disrupted in AD mouse models in the presence of phosphorylated tau and disorganized microtubules, or by the down-expression of AIS proteins like AnkyrinG or EB1, resulting in the missorting of hyperphosphorylated tau, which accumulates in the somatodendritic domain^24–26^.

In a previous study, a distal shift of the AIS was reported in hippocampal neurons from the tauopathy mouse model rTg4510^29^, although the neuronal content of phosphorylated tau in the neurons with an altered AIS was not directly evaluated. It is important to emphasize that, in the present study, we found heterogeneity in AIS alterations in AT8-immunopositive neurons in AD patients—with the most common changes being the proximal shift and lengthening of the AIS. It should be noted that similar changes were also found in tissue from non-demented human cases with tauopathy. Therefore, our results lead us to conclude that the accumulation of hyperphosphorylated tau is an important factor affecting AIS morphological features.

Various studies have demonstrated that the AIS is an efficient site to regulate neuronal activity as it has been shown to be subjected to plastic changes in response to neural activity^5,6,52^. Among the variety of AIS plasticity phenomena reported to date, it has been shown, in the chicken auditory system, that the loss of neuronal activity induced by the disruption of presynaptic sensory inputs leads to an increase in AIS length^6,53^. Visual information deprivation during the critical period of plasticity of visual cortical neurons prevents the shortening of the AIS and maintains a longer immature AIS^54^.

Alterations in neuronal excitability have been acknowledged as important features in AD^55^. Various studies in AD models have suggested important, albeit heterogeneous roles of tau in the modulation of neuronal excitability. For example, reduction of tau using antisense gene therapy or the removal of tau in knockout mice was reported to reduce hyperexcitability^56–58^. In addition, the presence of phosphorylated tau in the somatodendritic compartment has also been shown in vitro to decrease neuronal activity^27,28^. Furthermore, it has been shown—in the P301L tauopathy mouse model—that the effects induced by tau pathology on neuronal activity are heterogeneous and vary among different cortical regions. For instance, Rocher et al.^59^ reported that expression of mutated tau results in significant structural and functional changes in neurons, but that these changes were observed regardless of the presence of neurofibrillary tangles. In another study, a reduced action potential firing rate was reported in hyperphosphorylated tau hippocampal neurons^29^, whereas no functional changes were reported in the activity of neurons with neurofibrillary tangles in the visual cortex^60^.

It is known that changes in neuronal activity differentially affect the AIS of different neuronal populations. For example, neuronal chronic depolarization induces AIS shortening in dentate granule cells^61^ and an AIS distal shift in hippocampal CA1 pyramidal neurons^9^. Therefore, it is tempting to speculate that the heterogeneity in AIS changes found in the present study could be derived from differential changes in neuronal activity that the presence of hyperphosphorylated tau might induce in different neuronal populations. Further research is necessary to test this possibility. Finally, AIS relative position and length have been shown to modulate neuronal excitability^5,6^ with a negative linear relationship between action potential firing and the AIS distance to the soma^29^. Therefore, the heterogeneous changes in the AIS of different AT8-positive neurons found in the present study are likely to have a relevant impact on the excitability of these cells and could help to explain the different effects that the presence of pathological tau might induce in different neuronal populations. In summary, our results show that neurons with hyperphosphorylated tau aggregates undergo alterations in the length and/or in the position of the AIS, which are likely to alter normal neuronal excitability. This might contribute to neuronal dysfunction and to changes in the activity of neuronal circuits, contributing to the pathophysiology of AD. The various and complex causal relationships that might exist between phosphorylated tau accumulation, neuronal activity changes and AIS structural alterations—along with their potential variability between different populations—are interesting questions that remain to be clarified and warrant further research.

## Materials and methods

In the present study, we have used AD patient brain tissue obtained from the *Instituto de Neuropatología* (Dr. I. Ferrer, *Servicio de Anatomía Patológica*, IDIBELL-*Hospital Universitario de Bellvitge*, and Neurological Tissue Bank Biobanc-Hospital Clínic-IDIBAPS, Barcelona, Spain IF1, IF6, IF8, IF10, IF13, Bcn2, Bcn5, Bcn7, Bcn8, Bcn12 Bcn13 cases), as well as from the *Banco de Tejidos Fundación* CIEN (Dr. A. Rábano, *Área de Neuropatología, Centro Alzheimer, Fundación Reina Sofia*, Madrid, Spain; Vk11, BK16, Vk22, VK27 cases).Control human brain (AB1, AB5 and AB6) was obtained from the *Unidad Asociada Neuromax*—*Laboratorio de Neuroanatomía Humana, Facultad de Medicina, Universidad de Castilla-La Mancha*, Albacete and *Laboratorio Cajal de Circuitos Corticales UPM-CSIC*, Madrid, Spain). Following neuropathological examination, the AD stages were defined according to the Consortium to Establish a Registry for Alzheimer’s Disease (CERAD^41^) and the Braak and Braak criteria^40^.

Cases “AB1 and AB6” (Table 1) were considered as a type C1 control in our study, since individuals were free of neurological and psychiatric diseases, but have abundant AT8 positive cells in the cerebral cortex. The neurologic diagnosis for AB6 is III-A (Table 1). Unfortunately, there is no neurologic diagnosis according to Braak and Braak criteria for AB1, although NFTs are abundant in amygdala and hippocampus and extending slightly into the association cortex.

The postmortem delay in tissue obtained at autopsy, between death and tissue processing was below 6 h (Table 1), and the brain samples were obtained following the guidelines of the Institutional Ethical Committees, which also granted approval. Upon removal, the brains were immediately fixed in cold 4% paraformaldehyde in phosphate buffer (PB 0.1 M, pH7.4), and after 2 h, the tissue was cut into small blocks and post-fixed in the same fixative for 24–48 h at 4 °C. However, two human cases (AB1 and AB6) were intra-arterially perfused through the internal carotid artery 1 h after death with a saline solution followed by 4% paraformaldehyde in PB. The brain was then removed and post-fixed as described above. After fixation, it was immersed in graded sucrose solutions and stored in a cryoprotectant solution at − 20 °C. Serial Sections (50 μm) of the cortical tissue were obtained using a vibratome (St. Louis, MO, USA), and the sections from each region and case were batch-processed for immunohistochemical staining. The sections immediately adjacent to those stained immunohistochemically were Nissl-stained in order to identify the cortical areas and the laminar boundaries.

Despite having tried several immunocytochemical protocols, the combination of antibodies used for this study (see below) only rendered satisfactory results in tissue from 5 out of the 18 individual cases (see Table 1): three patients with AD (aged 75−91) and two control brain tissue (age 45 and 92) who were free of any known neurological or psychiatric illness.

### Immunofluorescence

For immunofluorescence, free floating serial sections from human brain tissue were first rinsed in PB and then pre-treated in 1.66% H_2_O_2_ for 30 min to inactivate the endogenous peroxidase activity. They were then preincubated for 1 h in PB with 0.25% Triton-X100 and 3% normal serum of the species in which the secondary antibodies were raised (Vector Laboratories, Burlingame, CA, USA). The sections were incubated for 48 h at 4 °C in the same stock solution containing the following primary antibodies: rabbit anti-βIV-Spectrin (1:1000, a gift from Dr. M. N. Rasband; Dept. of Neuroscience, Baylor College of Medicine, Houston, USA), guinea-pig anti-NeuN (Millipore, 1:2000) and mouse anti-phospho-PHF-tau pSer202 + Thr205 antibody (AT8, 1:2000, Pierce Endogen). After rinsing in PB, the sections were first incubated for 2 h at room temperature in biotinylated goat anti-rabbit antibody (1:200) to amplify the AIS immunoreactivity signal. Sections were then rinsed in PB and incubated for 2 h at room temperature in streptavidin-coupled Alexa 488 (1:200; Molecular Probes, Eugene, OR, USA), Alexa 594-conjugated goat anti-mouse and in Alexa 647-conjugated goat anti-guinea pig antibodies (1:1000; Molecular Probes, Eugene, OR, USA). After rinsing in PB, the sections were treated with Autofluorescence Eliminator Reagent (Chemicon; only human brain sections) to reduce autofluorescence. They were then mounted in antifade mounting medium (ProlongGold, Invitrogen) and studied with a Zeiss LSM 710 confocal laser scanning system (Carl Zeiss Microscopy GmbH, Jena, Germany).

### Image processing and statistics

At least four sections were examined (with a size of approximately 1 cm × 1cm × 50 µm) from each case. All the AT8-immunopositive cells (and adjacent AT8-immunonegative cells) present in each section, which AISs were fully visualized, were scanned. In total, we examined 164 AT8 + cells and 190 AT8- cells.

Confocal image stacks taken from the human temporal neocortex (areas 20/21/38 of Brodmann) were recorded at 0.14 µm intervals through separate channels with a 63 × oil-immersion lens (NA, 1.40, refraction index, 1.45). ZEN 2012 software (ZEN Digital Imaging for Light Microscopy, RRID:SCR_013672) was used to construct composite images from each optical series by combining the images recorded through the different channels (image resolution: 1024 × 1024 pixels; pixel size: 0.08 µm). Finally, we used a commercial software package (Imaris 6.4.0 Bitplane AG, Zurich, Switzerland) to measure—in 3D—the AIS length (measurement point tool) on the basis of βIV-Spectrin immunofluorescence. To establish AIS position, we measured the distance from the AIS start point and from the AIS end point from the edge of the soma, which was defined by NeuN immunofluorescence, present in both nuclei and cytoplasm and that extends into the proximal neuronal processes^62^. AIS length was calculated as the difference between AIS-start and AIS-end point distance values. To ensure that we selected complete AIS, we only considered those AIS, which had complete immunostaining separated in all dimensions from the edges of the image stacks by at least 5 µm.

Adobe Photoshop CS4 (Adobe Inc., 2019) software was used to compose figures. The differences in AIS length and distance from the soma between AT8 + and AT8 − immunostained cells were analyzed using a two-tailed paired t-test (BM Corp. Released 2016. IBM SPSS Statistics for Windows, Version 22.0. Armonk, NY: IBM Corp.).

### Ethical approval

Experiments with human tissue samples were performed following national laws and international ethical and technical guidelines on the use of human samples for biomedical research purposes. Written informed consent was obtained from all participants and/or their legal guardians and the approval of the whole donation process was obtained by local ethical committees (Comité de Bioseguridad IDIBELL, Comité de Ética de la Investigación del Instituto de Salud Carlos III and Comité de Ética BioBanc).

## Data Availability

The datasets used and/or analyzed during the current study are available from the corresponding author on reasonable request.
